# A large-scale profiling study of immune–coagulation associations in rheumatoid arthritis

**DOI:** 10.3389/fimmu.2026.1789560

**Published:** 2026-03-17

**Authors:** Ting Zhang, Fan Yang, Xiaohong Xu, Yanni Wang, Yunqing Zhang, Zhenglun Pan

**Affiliations:** Department of Rheumatology, Qilu Hospital (Qingdao), Cheeloo College of Medicine, Shandong University, Qingdao, China

**Keywords:** autoantibodies, biomarkers, complement, immune–coagulation, profiling

## Abstract

**Objective:**

To systematically profile associations between immunological markers and coagulation parameters in patients with rheumatoid arthritis (RA), and to evaluate the reproducibility and independence of these associations from demographic and inflammatory factors.

**Methods:**

We conducted a retrospective cross-sectional analysis of 1,656 hospitalized patients with RA. Associations between immunological markers (immunoglobulin G [IgG], immunoglobulin A [IgA], immunoglobulin M [IgM], rheumatoid factor [RF], anti–citrullinated protein antibodies [ACPA], C3, and C4) and coagulation parameters (international normalized ratio [INR], prothrombin time [PT], PT activity, activated partial thromboplastin time [APTT], D-dimer, and fibrinogen) were assessed using multivariable linear regression adjusted for age, sex, erythrocyte sedimentation rate, and high-sensitivity C-reactive protein. The cohort was divided into exploratory and validation subsets based on the median admission date. Robustness was examined using reduced-variable and principal component analysis–based sensitivity models.

**Results:**

Thirteen immune–coagulation associations were reproducibly identified across exploratory and validation cohorts. IgG showed coordinated associations with multiple coagulation parameters, including positive associations with INR, PT, APTT, and D-dimer, and negative associations with PT activity and fibrinogen. RF and ACPA exhibited inverse associations with APTT and fibrinogen, while complement components C3 and C4 were positively associated with fibrinogen levels. These patterns remained stable across sensitivity analyses, supporting the reproducibility of immune–coagulation coupling at the laboratory biomarker level.

**Conclusion:**

This large-scale profiling study delineates reproducible and internally validated associations between immunological markers and coagulation parameters in RA, revealing a structured immune–coagulation landscape at the laboratory level. Rather than implying clinical outcomes, these findings provide a reference framework to contextualize immune–coagulation interactions and support hypothesis-driven longitudinal or mechanistic investigations.

## Introduction

RA is a systemic autoimmune disease characterized by persistent immune activation that extends beyond joint inflammation and affects multiple biological pathways. Previous studies have reported an increased occurrence of coagulation abnormalities and thromboembolic events in RA (1, 2), prompting growing interest in immune–coagulation interactions within this disease context. These observations have stimulated investigation into how immune dysregulation may be reflected in coagulation-related laboratory phenotypes (3).

Recent studies have underscored the importance of the immuno-coagulative axis in the pathogenesis of RA, highlighting the interactions between autoantibodies, inflammatory cytokines, complement activation, endothelial dysfunction, and prothrombotic pathways (4). Notably, IgG glycosylation changes, RF, ACPA have been linked to neutrophil extracellular trap (NET) formation (5, 6), engaging Fcγ receptors, and amplifying tissue factor (TF) expression on endothelial cells, which have been reported to influence thrombin generation and fibrin deposition (7). Complement components such as C3 and C4 further amplify these processes by promoting neutrophil activation, endothelial injury, and hepatic acute-phase responses, including fibrinogen synthesis (8, 9).

Emerging mechanistic evidence suggests that these immune-coagulation interactions may contribute to both intravascular hypercoagulability and consumptive coagulopathy in RA. For example, elevated levels of D-dimer, PT, APTT, and INR, alongside reductions in fibrinogen and PT activity, have been observed in RA patients with active disease, reflecting simultaneous clot formation and depletion of clotting factors (10, 11). Furthermore, cytokines such as interleukin-6 (IL-6) and tumor necrosis factor-α (TNF-α) have been shown to synergistically upregulate proinflammatory and procoagulant pathways, linking immune activation to vascular injury and thrombogenesis (12, 13).

Despite increasing recognition of immune–coagulation crosstalk in RA, most prior studies have focused on individual pathways or specific biomarkers, often without systematic validation. Consequently, the structural relationships between circulating immunological markers and routinely measured coagulation parameters at the laboratory level remain incompletely characterized (14, 15). Addressing this gap is essential for establishing a reproducible biochemical reference framework that can support future longitudinal, mechanistic, or translational studies, rather than for immediate clinical prediction or risk stratification.

In this large retrospective study, we sought to systematically evaluate and validate the associations between a panel of immunological markers—including IgG, IgM, IgA, RF, ACPA, C3, and C4—and key coagulation parameters (INR, PT, PT activity, APTT, D-dimer, and fibrinogen) in RA patients. We additionally explored the independence of these associations from demographic (age, sex) and inflammatory (erythrocyte sedimentation rate [ESR] and high-sensitivity C-reactive protein [hsCRP]) covariates. By leveraging internal validation strategies and multi-model sensitivity analyses, our study provides a systematically validated characterization of immune–coagulation associations in RA and offers a laboratory-level framework to contextualize immune–coagulation interactions.

## Methods

### Study design and participants

**Figure 1:**
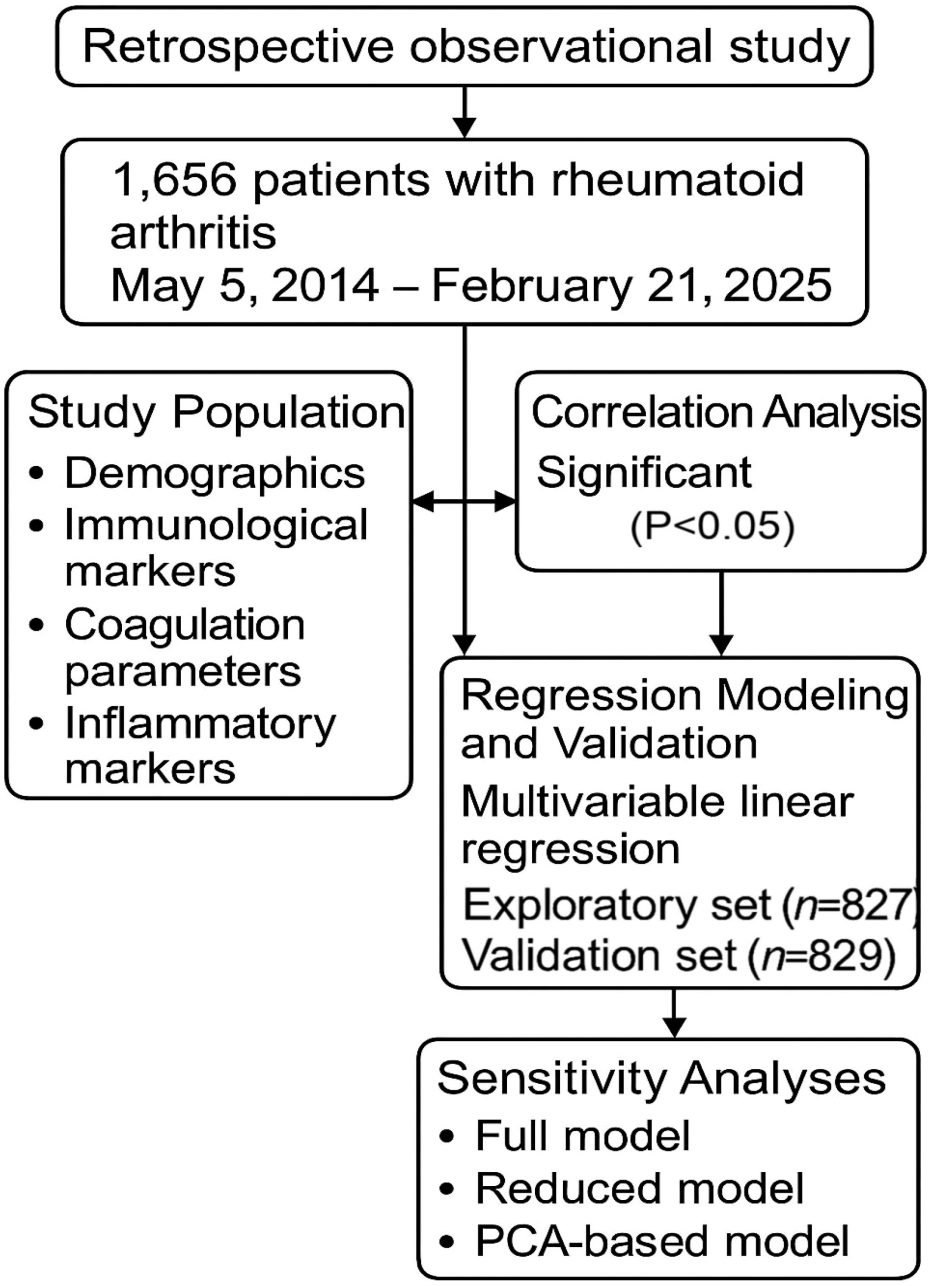
Study flowchart. Patients hospitalized with rheumatoid arthritis between May 5, 2014, and February 21, 2025, were included. Data cleaning and preprocessing were followed by cohort splitting at the median admission date (November 26, 2021) into an exploratory set (n = 827) and a validation set (n = 829). Multivariable regression analyses were performed in the exploratory set and validated in the independent validation set. Sensitivity analyses were conducted comparing the full model, reduced model, and PCA model.

This retrospective observational study included 1,656 patients diagnosed with RA based on the 2010 ACR/EULAR classification criteria and hospitalized at Qilu Hospital of Shandong University (Qingdao) between May 5, 2014, and February 21, 2025 (**Figure 1**). Demographic, immunological, inflammatory, and coagulation-related laboratory data were extracted from electronic medical records. Complete-case analyses were performed. Clarified exclusion of patients with missing key laboratory data.

### Laboratory markers

Immunological markers included RF, ACPA antibodies, IgG, IgA, IgM, C3 and C4. Coagulation parameters included INR, PT, PT activity, APTT, D-dimer, and fibrinogen. Inflammatory markers (covariates) included ESR and hsCRP. Gender and age were also recorded (**Table 1**).

**Table 1:** Baseline characteristics of the study population.

Variable	Mean (SD)	Median (IQR)
Age	58.030 (13.300)	60.000 (16.000)
RF	307.550 (752.300)	74.100 (258.650)
Anti_CCP	198.630 (173.650)	200.000 (343.300)
IgG	13.440 (3.800)	13.100 (4.100)
IgA	2.970 (1.300)	2.820 (1.470)
IgM	1.210 (0.710)	1.040 (0.680)
C3	1.150 (0.220)	1.130 (0.270)
C4	0.270 (0.080)	0.260 (0.090)
ESR	38.760 (26.490)	33.000 (39.750)
hsCRP	27.230 (36.960)	13.180 (32.260)
INR	1.030 (0.090)	1.030 (0.090)
PT	12.500 (1.310)	12.600 (1.800)
PT_Activity	97.900 (12.460)	97.940 (14.000)
APTT	34.540 (4.680)	34.130 (5.300)
D_dimer	1.670 (2.020)	1.050 (1.590)
Fibrinogen	3.960 (1.040)	3.930 (1.260)
Gender_Male	431 (26.000%)	

### Correlation analysis

Statistically significant correlations (P < 0.05) are summarized in **Supplementary Table 1**. Immunological markers were analyzed as continuous variables in original measurement unit. Pairwise correlations between immunological markers and coagulation parameters were assessed using Spearman correlation coefficients, given the non-normal distribution of several biomarkers. Correlation analyses were conducted for exploratory purposes to visualize the overall association structure and to inform the selection and interpretation of subsequent multivariable regression models. Correlation results were not used to infer independence, causality, or clinical relevance.

### Regression modeling and validation strategy

Multivariable linear regression was conducted to explore associations between immunological and coagulation markers, adjusting for age, gender, ESR, and hsCRP. To assess robustness, the cohort was divided by the median admission date (November 26, 2021) into an exploratory set (n = 827) and a validation set (n = 829) (**Figure 1**). Associations were first identified in the exploratory cohort using prespecified multivariable regression models (P < 0.05). The same model specifications were then applied unchanged to the validation cohort. No re-selection of variables or model refitting was performed in the validation set. Associations demonstrating consistent directionality and statistical significance across both cohorts were considered reproducible.

### Sensitivity analyses

To assess the potential influence of multicollinearity and model specification on the results, we performed sensitivity analyses using three modeling strategies (1): full model including all immunological markers (2); reduced model excluding variables with high variance inflation factor (VIF > 10) to minimize collinearity; and (3) PCA-based model using principal component analysis to reduce dimensionality and capture underlying immunological patterns (16). Model performance and stability were evaluated by comparing VIF, Akaike Information Criterion (AIC), and Bayesian Information Criterion (BIC) across models (17). The use of VIF and variable reduction is a standard approach to address collinearity in regression models (18). PCA offers an established technique for dimensionality reduction and capturing underlying data structures. The application of AIC and BIC for model comparison is widely recommended in statistical modeling. These strategies are consistent with established best practices in multivariable modeling and dimensionality reduction. Statistical significance was defined as a two-sided P-value < 0.05.

Ethics approval was obtained from the Institutional Review Board of Qilu Hospital of Shandong University. Given the retrospective nature of the study, informed consent was waived.

## Results

### Baseline characteristics

The mean age of the patients was 58.0 years (SD: 13.3), with 26% male. Median levels of RF and ACPA were elevated, consistent with active RA. Coagulation indices such as INR, PT, and D-dimer demonstrated mild elevations, while fibrinogen was within the high-normal range (**Table 1**).

### Correlation patterns

Significant pairwise correlations (P < 0.05) were observed between immune and coagulationmarkers (**Supplementary Table 1**, **Supplementary Figure 1**). Notably, IgG showed significant correlations with INR and PT, while RF and ACPA were correlated with APTT. C3 and C4 were strongly correlated with fibrinogen levels.

### Multivariable regression (full cohort)

In the full cohort (n = 1,656), multiple associations between immune and coagulation markers were significant after adjustment (**Table 2**). Detailed regression results across the full, exploratory, and validationcohorts—including all immunological markers and covariates—are provided in **Supplementary Table 2**. This comprehensive table offers insight into model consistency and effect direction across cohort subsets, supporting the robustness of the findings presented in **Table 2**. For example:IgG was positively associated with INR (β = 0.004, P = 3.6×10^-9^), PT (β = 0.063, P = 1.7×10^-11^), and D-dimer, and negatively associated with PT activity and fibrinogen. ACPA and RF were negatively associated with APTT. Complement C3 and C4 were positively associated with fibrinogen levels, consistent with inflammatory-reflecting hepatic synthesis.

**Table 2:** Multivariable regression analysis in the full cohort.

Outcome	Predictor	Beta	95% CI	P-value
INR	RF	-0.000	-0.000 to 0.000	0.065
INR	Anti_CCP	0.000	0.000 to 0.000	0.049
INR	IgG	0.004	0.002 to 0.005	3.605e-09
INR	IgA	0.003	-0.001 to 0.006	0.102
INR	IgM	0.000	-0.006 to 0.006	0.900
INR	C3	-0.061	-0.082 to -0.041	6.875e-09
INR	C4	-0.114	-0.164 to -0.064	8.129e-06
PT	RF	-0.000	-0.000 to 0.000	0.380
PT	Anti_CCP	-0.000	-0.001 to -0.000	0.019
PT	IgG	0.063	0.045 to 0.081	1.717e-11
PT	IgA	0.043	-0.010 to 0.096	0.114
PT	IgM	0.051	-0.040 to 0.142	0.273
PT	C3	-0.301	-0.620 to 0.017	0.064
PT	C4	-0.218	-0.987 to 0.551	0.578
PT_Activity	RF	0.001	-0.000 to 0.001	0.217
PT_Activity	Anti_CCP	-0.007	-0.011 to -0.004	2.289e-05
PT_Activity	IgG	-0.621	-0.787 to -0.454	4.743e-13
PT_Activity	IgA	-0.902	-1.386 to -0.418	0.000
PT_Activity	IgM	-0.070	-0.905 to 0.766	0.870
PT_Activity	C3	7.752	4.849 to 10.655	1.836e-07
PT_Activity	C4	11.538	4.497 to 18.579	0.001
APTT	RF	-0.001	-0.001 to -0.000	0.003
APTT	Anti_CCP	-0.004	-0.005 to -0.002	3.191e-08
APTT	IgG	0.172	0.106 to 0.238	3.415e-07
APTT	IgA	-0.020	-0.210 to 0.171	0.840
APTT	IgM	-0.025	-0.353 to 0.303	0.881
APTT	C3	-1.043	-2.191 to 0.105	0.075
APTT	C4	1.464	-1.308 to 4.237	0.300
D_dimer	RF	-0.000	-0.000 to -0.000	0.019
D_dimer	Anti_CCP	-0.001	-0.001 to 0.000	0.057
D_dimer	IgG	0.053	0.028 to 0.079	3.901e-05
D_dimer	IgA	-0.009	-0.082 to 0.064	0.815
D_dimer	IgM	-0.118	-0.243 to 0.008	0.066
D_dimer	C3	-0.334	-0.772 to 0.105	0.136
D_dimer	C4	-0.849	-1.907 to 0.210	0.116
Fibrinogen	RF	-0.000	-0.000 to -0.000	1.022e-08
Fibrinogen	Anti_CCP	-0.000	-0.000 to 0.000	0.939
Fibrinogen	IgG	-0.034	-0.044 to -0.024	1.595e-10
Fibrinogen	IgA	-0.049	-0.079 to -0.019	0.001
Fibrinogen	IgM	-0.108	-0.160 to -0.057	3.716e-05
Fibrinogen	C3	1.621	1.458 to 1.783	1.154e-76
Fibrinogen	C4	3.002	2.591 to 3.412	5.911e-44

### Adjusted vs. unadjusted models

**Figure 2:**
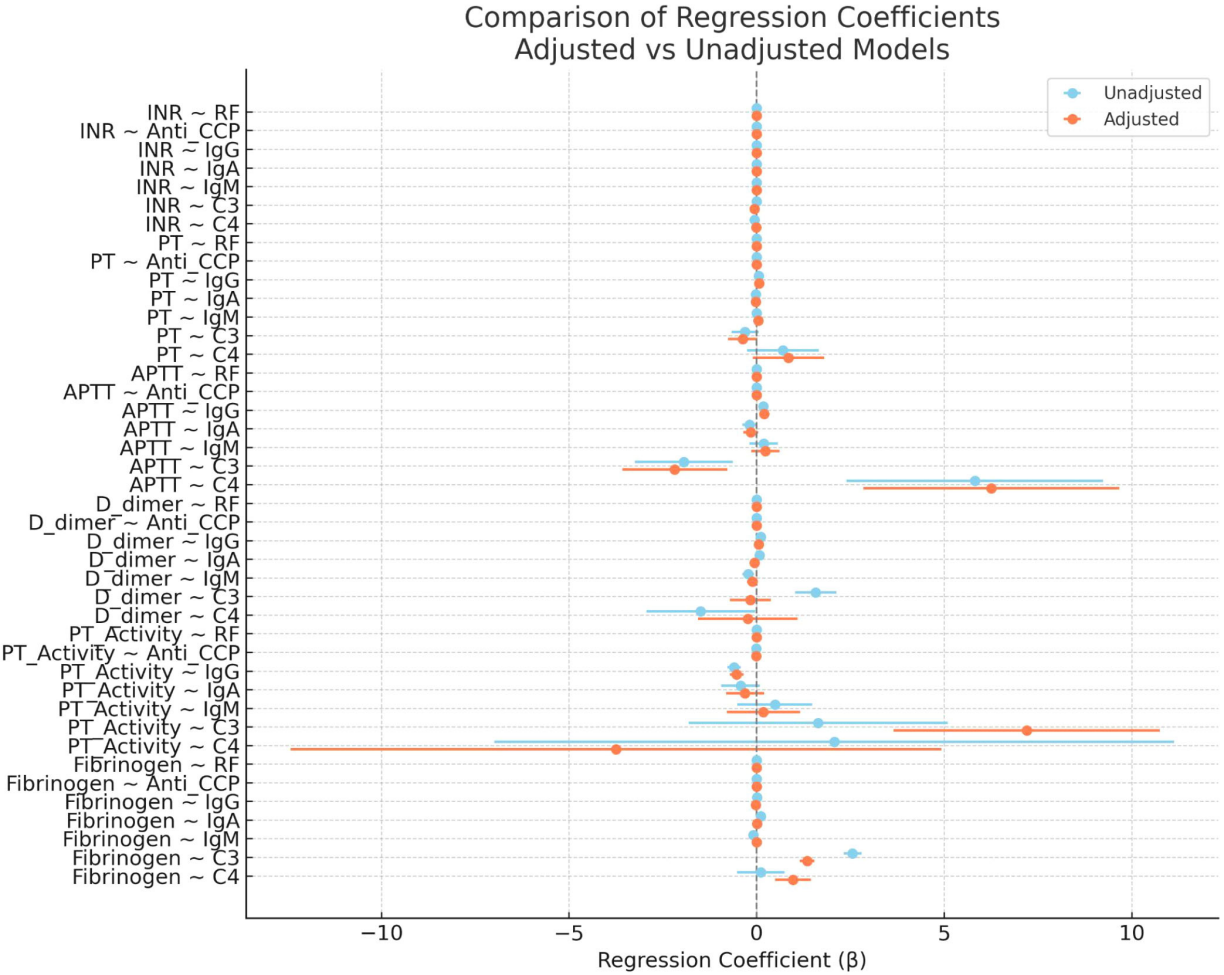
Comparison of regression coefficients for associations between immunological markers and coagulation parameters in rheumatoid arthritis, before and after adjustment for age, gender, ESR, and hsCRP. Each point represents the estimated regression coefficient (β) for a given immunological marker predicting a coagulation parameter, with horizontal lines indicating 95% confidence intervals. Blue markers denote unadjusted models, while orange markers denote adjusted models. A vertical dashed line at β = 0 indicates no association. Consistency in direction and statistical significance across models suggests independence from demographic and inflammatory confounders.

**Figure 2** compares coefficients before and after adjustment for demographic and inflammatoryvariables. Most validated associations, especially involving IgG and ACPA, showed consistentdirection and statistical significance after adjustment, indicating independence from age, sex, ESR, and hsCRP. For all immunological predictors, a side-by-side comparison of adjusted versus unadjusted regression coefficients is available in **Supplementary Table 3**. This table demonstrates that key associations, particularly those involving IgG and ACPA, remained stable after controlling for demographic and inflammatory covariates, reinforcing their independence.

### Internal validation of associations

**Figure 3:**
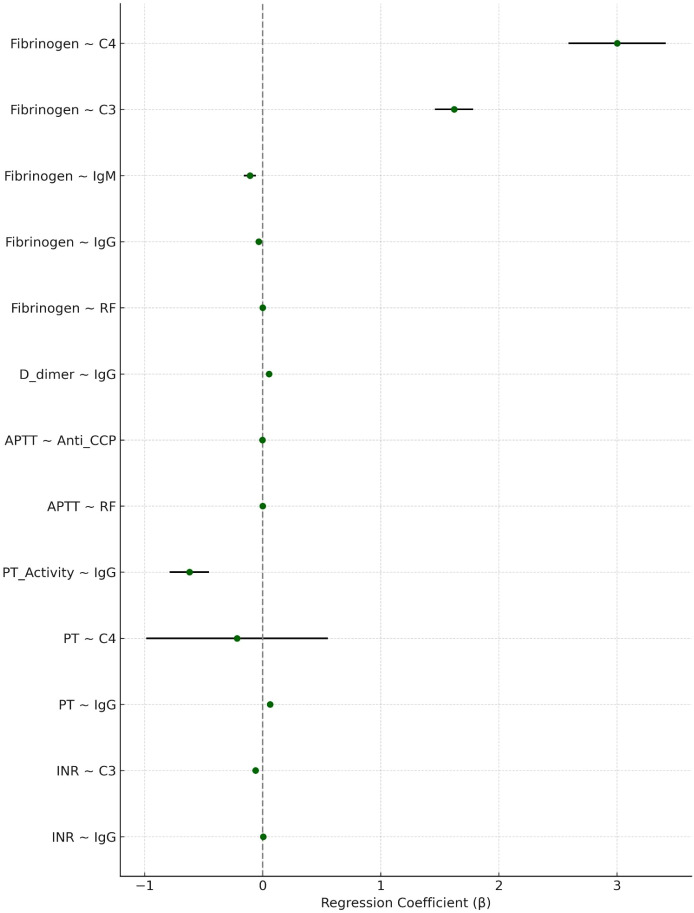
Forest plot of validated associations between immunological markers and coagulation parameters in patients with rheumatoid arthritis. Each point represents the regression coefficient (β) from multivariable linear regression adjusted for age, gender, ESR, and hsCRP, with horizontal lines indicating the corresponding 95% confidence intervals. Associations were validated by remaining statistically significant (P < 0.05) in both exploratory and validation sets.

Thirteen associations remained statistically significant in both the exploratory and validation sets (**Table 3**; **Figure 3**). These validated findings included:

**Table 3:** Validated associations between immunological markers and coagulation parameters after exploratory and validation analyses.

Outcome	Predictor	Beta (Full)	95% CI (Full)	P (Full)	Beta (Exploratory)	95% CI (Exploratory)	P (Exploratory)	Beta (Validation)	95% CI (Validation)	P (Validation)
INR	IgG	0.004	0.002 to 0.005	3.605e-09	0.003	0.001 to 0.004	0.001	0.005	0.003 to 0.007	2.737e-09
INR	C3	-0.061	-0.082 to -0.041	6.875e-09	-0.030	-0.059 to -0.001	0.042	-0.082	-0.110 to -0.053	2.572e-08
PT	IgG	0.063	0.045 to 0.081	1.717e-11	0.032	0.010 to 0.053	0.004	0.051	0.034 to 0.068	3.443e-09
PT	C4	-0.218	-0.987 to 0.551	0.578	-0.946	-1.863 to -0.029	0.043	-1.582	-2.266 to -0.897	6.630e-06
PT_Activity	IgG	-0.621	-0.787 to -0.454	4.743e-13	-0.479	-0.731 to -0.228	0.000	-0.779	-1.004 to -0.554	2.107e-11
APTT	RF	-0.001	-0.001 to -0.000	0.003	-0.000	-0.001 to -0.000	0.047	-0.001	-0.001 to -0.000	0.007
APTT	Anti_CCP	-0.004	-0.005 to -0.002	3.191e-08	-0.003	-0.004 to -0.002	2.871e-05	-0.004	-0.006 to -0.002	0.000
D_dimer	IgG	0.053	0.028 to 0.079	3.901e-05	0.052	0.008 to 0.096	0.021	0.065	0.037 to 0.092	3.490e-06
Fibrinogen	RF	-0.000	-0.000 to -0.000	1.022e-08	-0.000	-0.000 to -0.000	0.000	-0.000	-0.000 to -0.000	0.000
Fibrinogen	IgG	-0.034	-0.044 to -0.024	1.595e-10	-0.027	-0.041 to -0.013	0.000	-0.047	-0.060 to -0.033	7.689e-11
Fibrinogen	IgM	-0.108	-0.160 to -0.057	3.716e-05	-0.092	-0.156 to -0.027	0.006	-0.143	-0.219 to -0.068	0.000
Fibrinogen	C3	1.621	1.458 to 1.783	1.154e-76	1.330	1.099 to 1.561	1.209e-27	1.855	1.647 to 2.064	2.505e-58
Fibrinogen	C4	3.002	2.591 to 3.412	5.911e-44	2.164	1.562 to 2.767	3.918e-12	3.509	2.985 to 4.032	6.675e-36

IgG → INR↑, PT↑, APTT↑, D-dimer↑, Fibrinogen↓.
RF → APTT↓, Fibrinogen↓.
ACPA → APTT↓.
C3/C4 → Fibrinogen↑.
C4 → PT↓.

**Figure 4:**
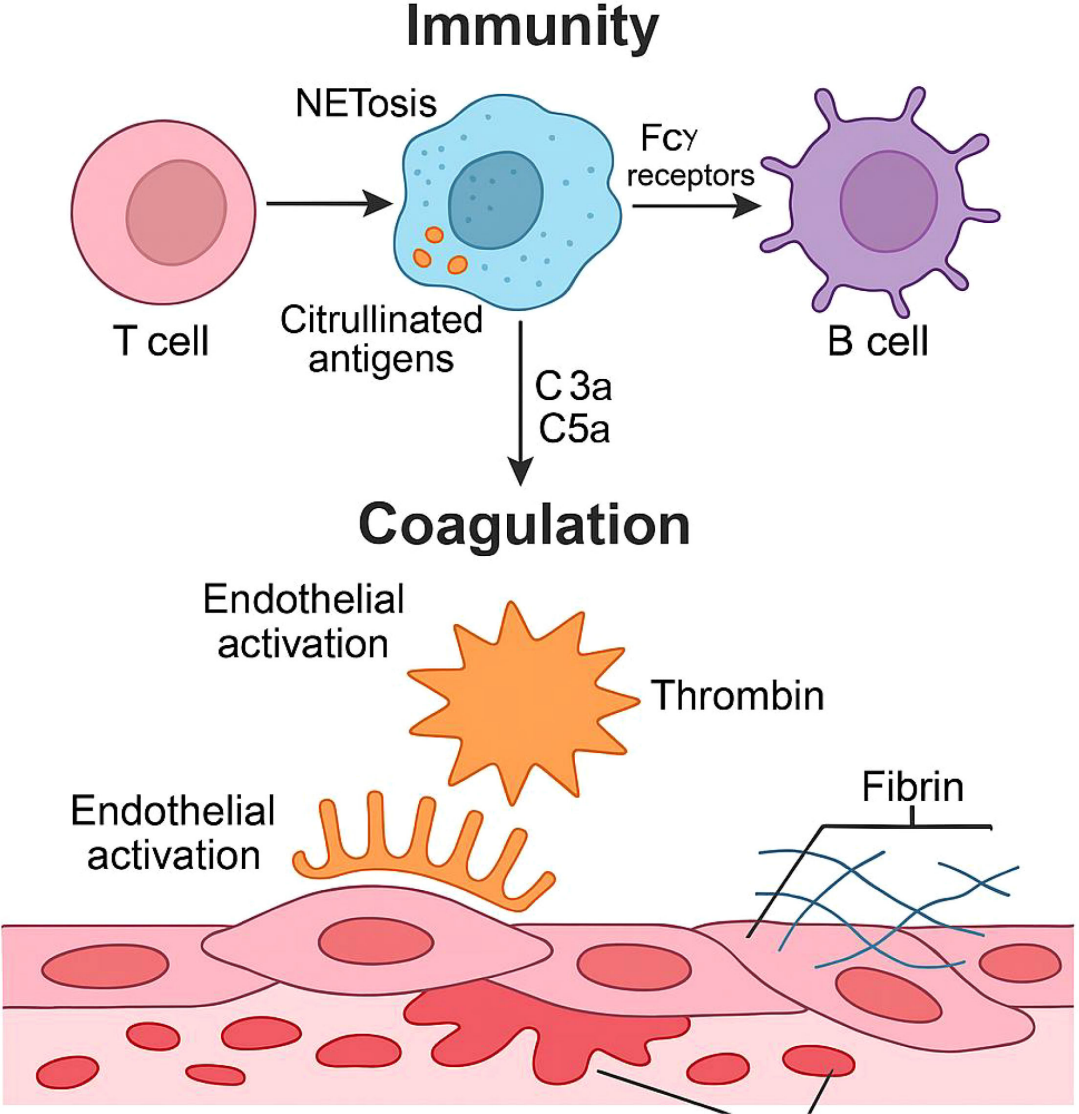
Conceptual framework illustrating literature-supported pathways linking humoral immune activity and coagulation-related biomarkers.

These associations support a robust immune–coagulation linkage in RA and informed the conceptual framework presented in this study (**Figure 4**).

### Sensitivity analyses

Validated associations retained their magnitude and direction across full, reduced, and PCA-basedmodels, underscoring the robustness of findings. (**Supplementary Table 4**; **Supplementary Figure 2**).

## Discussion

In this large retrospective cohort of patients with RA, we systematically profiled the associations between a comprehensive panel of immunological markers and routinely measured coagulation parameters. By applying an internal exploratory–validation framework and multiple sensitivity analyses, we identified a set of reproducible immune–coagulation associations that were largely robust to adjustment for demographic characteristics and systemic inflammatory markers. Importantly, this study was designed to delineate structured immune–coagulation coupling at the laboratory biomarker level rather than to assess clinical outcomes or prognostic relevance.

Among the immunological markers examined, IgG emerged as a central component within the immune–coagulation association network. IgG demonstrated coordinated associations with multiple coagulation indices, including INR, PT, APTT, D-dimer, PT activity, and fibrinogen, suggesting a broad and structured correspondence between humoral immune activity and coagulation-related laboratory parameters. IgG serves as a pivotal hub connecting inflammation, immune dysregulation, and thrombosis in rheumatoid arthritis (RA) through three synergistic mechanisms: immune complex (IC) load, Fcγ receptor (FcγR) signaling, and Fc-domain glycosylation. RA-specific autoantibodies (ACPA and RF) form highly proinflammatory ICs that deposit in joints and vascular endothelium, activating complement, inducing tissue factor expression, and disrupting coagulation-fibrinolysis balance. FcγR signaling networks (activatory FcγRI/IIIa vs. inhibitory FcγRIIB) regulate inflammation amplification and immune tolerance, with abnormal expression patterns exacerbating both joint damage and thrombotic risk. IgG Fc glycosylation (hypogalactosylation, hyposialylation in RA) acts as a molecular switch modulating pro/anti-inflammatory phenotypes and platelet-endothelial interactions. In addition, autoantibodies such as RF and ACPA exhibited distinct coagulation-related signatures, particularly inverse associations with APTT and fibrinogen. Complement components C3 and C4 showed strong and consistent positive associations with fibrinogen levels, reflecting their close relationship with acute-phase responses and hepatic protein synthesis. Collectively, these findings highlight that immune–coagulation interactions in RA are not uniform but display marker-specific and structured patterns.

From a mechanistic perspective, the observed immune–coagulation associations are consistent with prior experimental and translational studies describing immune–coagulation crosstalk in rheumatoid arthritis. Previous reports have shown that immunoglobulins, autoantibodies, and complement components can interact with endothelial cells, platelets, neutrophils, and hepatic pathways, thereby influencing coagulation-related biological processes (19–23). In particular, IgG-containing immune complexes and autoantibodies such as RF and ACPA have been linked to Fcγ receptor engagement and neutrophil activation, while complement pathways have been implicated in modulating coagulation factor regulation and acute-phase responses (24–28). Within this literature-based framework, the patterns identified in the present study are compatible with coordinated immune–coagulation coupling at the laboratory biomarker level. Importantly, these associations should be interpreted as biochemical correspondences rather than evidence of specific cellular events or pathogenic mechanisms, given the absence of functional or longitudinal data.

The robustness of these laboratory-level associations was further supported by sensitivity analyses using reduced-variable and principal component analysis (PCA)-based models, minimizing the likelihood that the observed patterns were driven by multicollinearity or model overfitting. Importantly, given the cross-sectional design and the absence of clinical thrombotic or longitudinal outcome data, our findings should not be interpreted as evidence of thrombotic risk or prognostic relevance. Instead, they provide a reproducible biochemical profiling of how immunoglobulins, autoantibodies, and complement components correspond to coagulation-related laboratory parameters. This profiling framework may help contextualize immune–coagulation interactions in RA and supports the rationale for future studies integrating longitudinal outcomes, functional assays, or interventional designs, where clinical relevance can be directly assessed.

Several strengths enhance the interpretability of this study, including the large sample size, systematic evaluation of multiple immune and coagulation markers, internal validation, and consistent findings across multiple sensitivity analyses. Nevertheless, important limitations should be acknowledged. The retrospective and cross-sectional nature of the analysis precludes causal inference, and the lack of clinical outcome data limits interpretation to laboratory-level associations. Due to incomplete and non-uniform documentation of treatment exposure in this retrospective dataset, adjustment for medication effects was not feasible. The absence of detailed medication data (including corticosteroids, csDMARDs, and biologics) represents a major limitation. Glucocorticoids directly inhibit B cell function, significantly reducing the synthesis and secretion of IgG, IgA, IgM, and autoantibodies (RF, ACPA). Different DMARD classes (e.g., methotrexate, biological agents) downregulate immune markers to varying degrees via targeted modulation of immune pathways (e.g., TNF-α, IL-6 signaling). These pharmacologic effects may directly compromise the validity of observed immune-coagulation associations. Glucocorticoids enhance procoagulant activity by promoting hepatic synthesis of fibrinogen and coagulation factors. In contrast, biological agents and csDMARDs reduce fibrinogen and D-dimer levels and modulate coagulation parameters (e.g., PT, APTT) via inflammation attenuation. Direct or indirect pharmacologic regulation of coagulation markers may mask or exaggerate intrinsic associations between immune biomarkers and coagulation parameters. Treatment regimen selection is strongly associated with disease activity, duration, comorbidities, and other factors that independently alter immune and coagulation function. Without detailed medication data, it is impossible to delineate whether immune-coagulation associations arise from the disease’s intrinsic pathophysiology, are mediated by therapeutic interventions, or reflect their combined effects. This ambiguity may introduce bias into association results, limiting the accuracy and generalizability of conclusions. In addition, the cohort consists of hospitalized RA patients. Associations may be accentuated under heightened inflammatory conditions. Findings may not be directly generalizable to stable outpatient RA populations. Results are most applicable to hospitalized or high-activity RA settings.

In summary, this study provides a comprehensive and internally validated profiling of immune–coagulation associations in rheumatoid arthritis at the laboratory biomarker level. By delineating a structured immuno–coagulative landscape, our findings offer a reference framework for hypothesis-driven longitudinal, functional, or interventional investigations, rather than for direct clinical inference or risk prediction.

## Data Availability

The original contributions presented in the study are included in the article/**Supplementary Material**. Further inquiries can be directed to the corresponding author.
