# Supplementary material for: A large-scale profiling study of immune–coagulation associations in rheumatoid arthritis
Source: Front Immunol. 2026 Mar 17;17:1789560. doi: 10.3389/fimmu.2026.1789560 (PMC13047912; doi:10.3389/fimmu.2026.1789560)
Supplement: Supplementary file 1 [file Table1.docx]

# Supplementary Tables

Supplementary Table 1. Pairwise correlation analysis between immunological markers and coagulation parameters in the full cohort.

|  | RF | Anti_CCP | IgG | IgA | IgM | C3 | C4 | INR | PT | APTT |
| --- | --- | --- | --- | --- | --- | --- | --- | --- | --- | --- |
| RF | 0.000 | 2.991e-60 | 0.001 | 6.014e-16 | 3.722e-64 | 0.005 | 0.119 | 0.618 | 0.626 | 1.179e-05 |
| Anti_CCP | 2.991e-60 | 0.000 | 0.000 | 1.816e-06 | 8.293e-06 | 0.917 | 0.377 | 0.036 | 0.002 | 4.460e-08 |
| IgG | 0.001 | 0.000 | 0.000 | 2.826e-78 | 1.652e-18 | 0.001 | 2.558e-05 | 4.234e-14 | 9.287e-07 | 0.012 |
| IgA | 6.014e-16 | 1.816e-06 | 2.826e-78 | 0.000 | 9.792e-15 | 3.635e-05 | 0.425 | 2.693e-06 | 0.342 | 0.185 |
| IgM | 3.722e-64 | 8.293e-06 | 1.652e-18 | 9.792e-15 | 0.000 | 0.001 | 0.000 | 0.280 | 0.498 | 0.585 |
| C3 | 0.005 | 0.917 | 0.001 | 3.635e-05 | 0.001 | 0.000 | 2.721e-134 | 0.513 | 0.759 | 0.517 |
| C4 | 0.119 | 0.377 | 2.558e-05 | 0.425 | 0.000 | 2.721e-134 | 0.000 | 0.010 | 0.793 | 0.047 |
| INR | 0.618 | 0.036 | 4.234e-14 | 2.693e-06 | 0.280 | 0.513 | 0.010 | 0.000 | 1.295e-19 | 0.149 |
| PT | 0.626 | 0.002 | 9.287e-07 | 0.342 | 0.498 | 0.759 | 0.793 | 1.295e-19 | 0.000 | 2.022e-142 |
| APTT | 1.179e-05 | 4.460e-08 | 0.012 | 0.185 | 0.585 | 0.517 | 0.047 | 0.149 | 2.022e-142 | 0.000 |

*Spearman correlation coefficients and corresponding P values are shown. Spearman methods were applied due to non-normal distributions of several biomarkers. Correlation analyses are exploratory and were used to guide subsequent multivariable regression modeling.

Supplementary Table 2. Multivariable linear regression analyses examining associations between immunological markers and coagulation parameters in the full cohort, exploratory cohort, and validation cohort.

| Cohort | Outcome | Variable | Beta | 95% CI | P-value |
| --- | --- | --- | --- | --- | --- |
| Full | INR | const | 1.063 | 1.028 to 1.098 | 0.0 |
| Full | INR | RF | 0.0 | -0.0 to 0.0 | 0.146 |
| Full | INR | Anti_CCP | 0.0 | -0.0 to 0.0 | 0.0802 |
| Full | INR | IgG | 0.003 | 0.002 to 0.004 | 2.569e-06 |
| Full | INR | IgA | -0.001 | -0.004 to 0.003 | 0.7054 |
| Full | INR | IgM | 0.001 | -0.006 to 0.008 | 0.8524 |
| Full | INR | C3 | -0.05 | -0.076 to -0.025 | 0.0001093 |
| Full | INR | C4 | -0.014 | -0.076 to 0.048 | 0.6541 |
| Full | INR | Age | -0.001 | -0.001 to -0.0 | 8.074e-06 |
| Full | INR | Gender_Male | 0.023 | 0.014 to 0.032 | 1.871e-06 |
| Full | INR | ESR | 0.0 | -0.0 to 0.0 | 0.1059 |
| Full | INR | hsCRP | 0.001 | 0.001 to 0.001 | 1.026e-21 |
| Full | PT | const | 11.868 | 11.329 to 12.408 | 2.905e-271 |
| Full | PT | RF | 0.0 | -0.0 to 0.0 | 0.405 |
| Full | PT | Anti_CCP | 0.0 | -0.001 to -0.0 | 0.007308 |
| Full | PT | IgG | 0.066 | 0.046 to 0.086 | 1.192e-10 |
| Full | PT | IgA | -0.016 | -0.072 to 0.04 | 0.5728 |
| Full | PT | IgM | 0.045 | -0.062 to 0.152 | 0.4131 |
| Full | PT | C3 | -0.365 | -0.756 to 0.025 | 0.06678 |
| Full | PT | C4 | 0.85 | -0.104 to 1.805 | 0.08086 |
| Full | PT | Age | 0.001 | -0.003 to 0.006 | 0.5601 |
| Full | PT | Gender_Male | 0.174 | 0.028 to 0.319 | 0.01908 |
| Full | PT | ESR | -0.007 | -0.01 to -0.003 | 0.0002415 |
| Full | PT | hsCRP | 0.007 | 0.005 to 0.009 | 1.149e-09 |
| Full | PT_Activity | const | 97.543 | 92.637 to 102.448 | 4.642e-235 |
| Full | PT_Activity | RF | 0.0 | -0.0 to 0.001 | 0.3461 |
| Full | PT_Activity | Anti_CCP | -0.007 | -0.01 to -0.003 | 8.852e-05 |
| Full | PT_Activity | IgG | -0.53 | -0.711 to -0.349 | 1.049e-08 |
| Full | PT_Activity | IgA | -0.307 | -0.817 to 0.203 | 0.2381 |
| Full | PT_Activity | IgM | 0.184 | -0.79 to 1.158 | 0.7111 |
| Full | PT_Activity | C3 | 7.2 | 3.649 to 10.751 | 7.286e-05 |
| Full | PT_Activity | C4 | -3.745 | -12.425 to 4.934 | 0.3974 |
| Full | PT_Activity | Age | 0.099 | 0.054 to 0.144 | 1.596e-05 |
| Full | PT_Activity | Gender_Male | -2.887 | -4.205 to -1.568 | 1.868e-05 |
| Full | PT_Activity | ESR | -0.006 | -0.038 to 0.027 | 0.7273 |
| Full | PT_Activity | hsCRP | -0.1 | -0.12 to -0.08 | 1.4129999999999999e-22 |
| Full | APTT | const | 34.827 | 32.897 to 36.756 | 9.998999999999999e-204 |
| Full | APTT | RF | -0.001 | -0.001 to -0.0 | 0.006165 |
| Full | APTT | Anti_CCP | -0.004 | -0.005 to -0.002 | 1.369e-08 |
| Full | APTT | IgG | 0.202 | 0.131 to 0.274 | 2.771e-08 |
| Full | APTT | IgA | -0.153 | -0.354 to 0.048 | 0.1347 |
| Full | APTT | IgM | 0.236 | -0.146 to 0.619 | 0.226 |
| Full | APTT | C3 | -2.18 | -3.577 to -0.783 | 0.002238 |
| Full | APTT | C4 | 6.255 | 2.841 to 9.669 | 0.000336 |
| Full | APTT | Age | -0.023 | -0.041 to -0.005 | 0.01052 |
| Full | APTT | Gender_Male | 1.037 | 0.518 to 1.555 | 9.242e-05 |
| Full | APTT | ESR | -0.01 | -0.023 to 0.002 | 0.1096 |
| Full | APTT | hsCRP | 0.014 | 0.006 to 0.022 | 0.0003247 |
| Full | D_dimer | const | -0.004 | -0.753 to 0.746 | 0.9922 |
| Full | D_dimer | RF | 0.0 | -0.0 to 0.0 | 0.4103 |
| Full | D_dimer | Anti_CCP | -0.001 | -0.001 to -0.0 | 0.04796 |
| Full | D_dimer | IgG | 0.061 | 0.033 to 0.088 | 1.784e-05 |
| Full | D_dimer | IgA | -0.05 | -0.128 to 0.028 | 0.2114 |
| Full | D_dimer | IgM | -0.111 | -0.26 to 0.038 | 0.1437 |
| Full | D_dimer | C3 | -0.166 | -0.708 to 0.377 | 0.5495 |
| Full | D_dimer | C4 | -0.23 | -1.556 to 1.096 | 0.7336 |
| Full | D_dimer | Age | 0.006 | -0.001 to 0.013 | 0.09853 |
| Full | D_dimer | Gender_Male | 0.063 | -0.139 to 0.264 | 0.5428 |
| Full | D_dimer | ESR | 0.02 | 0.015 to 0.025 | 9.417e-15 |
| Full | D_dimer | hsCRP | 0.015 | 0.012 to 0.018 | 2.9e-21 |
| Full | Fibrinogen | const | 1.251 | 0.978 to 1.524 | 6.658e-19 |
| Full | Fibrinogen | RF | 0.0 | -0.0 to -0.0 | 1.408e-09 |
| Full | Fibrinogen | Anti_CCP | 0.0 | -0.0 to 0.0 | 0.4891 |
| Full | Fibrinogen | IgG | -0.021 | -0.031 to -0.011 | 4.182e-05 |
| Full | Fibrinogen | IgA | 0.007 | -0.021 to 0.036 | 0.6123 |
| Full | Fibrinogen | IgM | 0.002 | -0.052 to 0.056 | 0.9518 |
| Full | Fibrinogen | C3 | 1.347 | 1.15 to 1.545 | 9.052e-39 |
| Full | Fibrinogen | C4 | 0.97 | 0.487 to 1.453 | 8.52e-05 |
| Full | Fibrinogen | Age | 0.005 | 0.002 to 0.007 | 0.0002556 |
| Full | Fibrinogen | Gender_Male | 0.293 | 0.22 to 0.367 | 8.254e-15 |
| Full | Fibrinogen | ESR | 0.017 | 0.015 to 0.019 | 9.310000000000001e-69 |
| Full | Fibrinogen | hsCRP | 0.008 | 0.007 to 0.009 | 6.509e-40 |
| Exploratory | INR | const | 1.027 | 0.979 to 1.076 | 2.107e-202 |
| Exploratory | INR | RF | 0.0 | -0.0 to 0.0 | 0.5607 |
| Exploratory | INR | Anti_CCP | 0.0 | 0.0 to 0.0 | 0.001941 |
| Exploratory | INR | IgG | 0.004 | 0.002 to 0.006 | 7.492e-06 |
| Exploratory | INR | IgA | 0.001 | -0.003 to 0.006 | 0.5595 |
| Exploratory | INR | IgM | 0.002 | -0.008 to 0.012 | 0.6771 |
| Exploratory | INR | C3 | -0.052 | -0.086 to -0.018 | 0.002677 |
| Exploratory | INR | C4 | 0.005 | -0.076 to 0.086 | 0.9029 |
| Exploratory | INR | Age | -0.001 | -0.001 to -0.0 | 0.01205 |
| Exploratory | INR | Gender_Male | 0.009 | -0.005 to 0.022 | 0.216 |
| Exploratory | INR | ESR | 0.0 | -0.0 to 0.0 | 0.5752 |
| Exploratory | INR | hsCRP | 0.001 | 0.001 to 0.001 | 3.094e-16 |
| Exploratory | PT | const | 11.476 | 10.773 to 12.179 | 7.32e-146 |
| Exploratory | PT | RF | 0.0 | -0.0 to 0.0 | 0.7331 |
| Exploratory | PT | Anti_CCP | -0.001 | -0.001 to -0.0 | 0.001131 |
| Exploratory | PT | IgG | 0.087 | 0.061 to 0.112 | 2.894e-11 |
| Exploratory | PT | IgA | -0.038 | -0.11 to 0.034 | 0.2984 |
| Exploratory | PT | IgM | 0.025 | -0.122 to 0.172 | 0.7384 |
| Exploratory | PT | C3 | -0.199 | -0.693 to 0.295 | 0.4297 |
| Exploratory | PT | C4 | 1.244 | 0.066 to 2.422 | 0.0385 |
| Exploratory | PT | Age | 0.003 | -0.003 to 0.01 | 0.3111 |
| Exploratory | PT | Gender_Male | 0.302 | 0.104 to 0.499 | 0.002852 |
| Exploratory | PT | ESR | -0.006 | -0.011 to -0.001 | 0.02132 |
| Exploratory | PT | hsCRP | 0.005 | 0.002 to 0.008 | 0.0003589 |
| Exploratory | PT_Activity | const | 101.18 | 94.119 to 108.24 | 8.615000000000001e-122 |
| Exploratory | PT_Activity | RF | 0.0 | -0.002 to 0.002 | 0.8996 |
| Exploratory | PT_Activity | Anti_CCP | -0.01 | -0.015 to -0.005 | 9.192e-05 |
| Exploratory | PT_Activity | IgG | -0.65 | -0.903 to -0.397 | 5.916e-07 |
| Exploratory | PT_Activity | IgA | -0.463 | -1.183 to 0.257 | 0.2069 |
| Exploratory | PT_Activity | IgM | -0.105 | -1.582 to 1.372 | 0.8888 |
| Exploratory | PT_Activity | C3 | 7.622 | 2.659 to 12.585 | 0.002654 |
| Exploratory | PT_Activity | C4 | -4.716 | -16.545 to 7.113 | 0.4341 |
| Exploratory | PT_Activity | Age | 0.078 | 0.012 to 0.144 | 0.02099 |
| Exploratory | PT_Activity | Gender_Male | -0.989 | -2.976 to 0.997 | 0.3286 |
| Exploratory | PT_Activity | ESR | 0.025 | -0.024 to 0.074 | 0.3115 |
| Exploratory | PT_Activity | hsCRP | -0.125 | -0.155 to -0.094 | 1.889e-15 |
| Exploratory | APTT | const | 34.118 | 31.202 to 37.034 | 3.667e-90 |
| Exploratory | APTT | RF | -0.001 | -0.001 to 0.0 | 0.06377 |
| Exploratory | APTT | Anti_CCP | -0.005 | -0.007 to -0.003 | 1.383e-07 |
| Exploratory | APTT | IgG | 0.226 | 0.121 to 0.33 | 2.583e-05 |
| Exploratory | APTT | IgA | -0.243 | -0.541 to 0.054 | 0.1087 |
| Exploratory | APTT | IgM | 0.348 | -0.262 to 0.958 | 0.2635 |
| Exploratory | APTT | C3 | -1.972 | -4.021 to 0.078 | 0.05939 |
| Exploratory | APTT | C4 | 6.58 | 1.694 to 11.465 | 0.008364 |
| Exploratory | APTT | Age | -0.012 | -0.04 to 0.015 | 0.3764 |
| Exploratory | APTT | Gender_Male | 2.134 | 1.313 to 2.954 | 4.133e-07 |
| Exploratory | APTT | ESR | -0.003 | -0.023 to 0.018 | 0.8062 |
| Exploratory | APTT | hsCRP | 0.011 | -0.001 to 0.024 | 0.07643 |
| Exploratory | D_dimer | const | -0.423 | -1.479 to 0.633 | 0.4317 |
| Exploratory | D_dimer | RF | 0.0 | -0.0 to 0.0 | 0.9066 |
| Exploratory | D_dimer | Anti_CCP | 0.0 | -0.001 to 0.0 | 0.3452 |
| Exploratory | D_dimer | IgG | 0.095 | 0.057 to 0.133 | 1.114e-06 |
| Exploratory | D_dimer | IgA | -0.035 | -0.143 to 0.072 | 0.5206 |
| Exploratory | D_dimer | IgM | -0.106 | -0.327 to 0.115 | 0.3478 |
| Exploratory | D_dimer | C3 | -0.234 | -0.977 to 0.508 | 0.5354 |
| Exploratory | D_dimer | C4 | -0.238 | -2.007 to 1.531 | 0.7915 |
| Exploratory | D_dimer | Age | 0.003 | -0.007 to 0.013 | 0.5373 |
| Exploratory | D_dimer | Gender_Male | -0.065 | -0.362 to 0.232 | 0.6657 |
| Exploratory | D_dimer | ESR | 0.019 | 0.012 to 0.027 | 2.588e-07 |
| Exploratory | D_dimer | hsCRP | 0.019 | 0.014 to 0.023 | 2.326e-15 |
| Exploratory | Fibrinogen | const | 1.142 | 0.784 to 1.501 | 6.701e-10 |
| Exploratory | Fibrinogen | RF | 0.0 | -0.0 to -0.0 | 0.01418 |
| Exploratory | Fibrinogen | Anti_CCP | 0.0 | -0.0 to 0.0 | 0.4894 |
| Exploratory | Fibrinogen | IgG | -0.019 | -0.032 to -0.006 | 0.004488 |
| Exploratory | Fibrinogen | IgA | -0.005 | -0.041 to 0.032 | 0.7937 |
| Exploratory | Fibrinogen | IgM | -0.029 | -0.104 to 0.046 | 0.4513 |
| Exploratory | Fibrinogen | C3 | 1.359 | 1.106 to 1.611 | 1.51e-24 |
| Exploratory | Fibrinogen | C4 | 1.024 | 0.423 to 1.625 | 0.0008678 |
| Exploratory | Fibrinogen | Age | 0.004 | 0.001 to 0.008 | 0.009533 |
| Exploratory | Fibrinogen | Gender_Male | 0.279 | 0.178 to 0.38 | 7.617e-08 |
| Exploratory | Fibrinogen | ESR | 0.018 | 0.016 to 0.021 | 6.676e-43 |
| Exploratory | Fibrinogen | hsCRP | 0.01 | 0.008 to 0.011 | 2.6639999999999997e-33 |
| Validation | INR | const | 1.102 | 1.051 to 1.152 | 7.663e-208 |
| Validation | INR | RF | 0.0 | -0.0 to 0.0 | 0.2433 |
| Validation | INR | Anti_CCP | 0.0 | -0.0 to 0.0 | 0.6671 |
| Validation | INR | IgG | 0.002 | -0.0 to 0.004 | 0.06217 |
| Validation | INR | IgA | -0.002 | -0.008 to 0.003 | 0.3781 |
| Validation | INR | IgM | 0.0 | -0.009 to 0.01 | 0.9608 |
| Validation | INR | C3 | -0.047 | -0.085 to -0.009 | 0.01473 |
| Validation | INR | C4 | -0.047 | -0.142 to 0.048 | 0.3292 |
| Validation | INR | Age | -0.001 | -0.001 to -0.0 | 0.000528 |
| Validation | INR | Gender_Male | 0.032 | 0.019 to 0.045 | 1.594e-06 |
| Validation | INR | ESR | 0.0 | 0.0 to 0.001 | 0.01826 |
| Validation | INR | hsCRP | 0.001 | 0.0 to 0.001 | 8.339e-09 |
| Validation | PT | const | 12.462 | 11.649 to 13.274 | 9.842e-134 |
| Validation | PT | RF | 0.0 | -0.0 to 0.0 | 0.312 |
| Validation | PT | Anti_CCP | 0.0 | -0.001 to 0.0 | 0.3723 |
| Validation | PT | IgG | 0.048 | 0.017 to 0.078 | 0.002477 |
| Validation | PT | IgA | 0.005 | -0.081 to 0.09 | 0.9137 |
| Validation | PT | IgM | 0.022 | -0.133 to 0.177 | 0.7819 |
| Validation | PT | C3 | -0.529 | -1.132 to 0.074 | 0.08554 |
| Validation | PT | C4 | 0.294 | -1.222 to 1.809 | 0.7038 |
| Validation | PT | Age | -0.004 | -0.011 to 0.003 | 0.2858 |
| Validation | PT | Gender_Male | 0.121 | -0.088 to 0.33 | 0.2559 |
| Validation | PT | ESR | -0.006 | -0.011 to -0.001 | 0.02162 |
| Validation | PT | hsCRP | 0.007 | 0.004 to 0.01 | 3.73e-06 |
| Validation | PT_Activity | const | 93.375 | 86.484 to 100.266 | 3.815e-112 |
| Validation | PT_Activity | RF | 0.0 | -0.001 to 0.002 | 0.4621 |
| Validation | PT_Activity | Anti_CCP | -0.004 | -0.008 to 0.001 | 0.0956 |
| Validation | PT_Activity | IgG | -0.378 | -0.639 to -0.117 | 0.004608 |
| Validation | PT_Activity | IgA | -0.192 | -0.919 to 0.534 | 0.603 |
| Validation | PT_Activity | IgM | 0.437 | -0.875 to 1.749 | 0.5135 |
| Validation | PT_Activity | C3 | 6.827 | 1.709 to 11.945 | 0.008998 |
| Validation | PT_Activity | C4 | -1.486 | -14.341 to 11.369 | 0.8206 |
| Validation | PT_Activity | Age | 0.117 | 0.055 to 0.179 | 0.0002172 |
| Validation | PT_Activity | Gender_Male | -4.209 | -5.984 to -2.435 | 3.754e-06 |
| Validation | PT_Activity | ESR | -0.028 | -0.071 to 0.015 | 0.2052 |
| Validation | PT_Activity | hsCRP | -0.082 | -0.108 to -0.056 | 1.359e-09 |
| Validation | APTT | const | 35.699 | 33.193 to 38.205 | 1.62e-120 |
| Validation | APTT | RF | 0.0 | -0.001 to -0.0 | 0.04489 |
| Validation | APTT | Anti_CCP | -0.002 | -0.004 to -0.001 | 0.004786 |
| Validation | APTT | IgG | 0.184 | 0.089 to 0.279 | 0.0001559 |
| Validation | APTT | IgA | -0.075 | -0.339 to 0.189 | 0.5759 |
| Validation | APTT | IgM | 0.089 | -0.389 to 0.566 | 0.7155 |
| Validation | APTT | C3 | -2.226 | -4.087 to -0.365 | 0.01913 |
| Validation | APTT | C4 | 5.862 | 1.187 to 10.537 | 0.01405 |
| Validation | APTT | Age | -0.041 | -0.063 to -0.018 | 0.0004345 |
| Validation | APTT | Gender_Male | 0.253 | -0.392 to 0.899 | 0.4413 |
| Validation | APTT | ESR | -0.013 | -0.029 to 0.002 | 0.09338 |
| Validation | APTT | hsCRP | 0.014 | 0.005 to 0.024 | 0.00318 |
| Validation | D_dimer | const | 0.621 | -0.451 to 1.693 | 0.2558 |
| Validation | D_dimer | RF | 0.0 | -0.0 to 0.0 | 0.5476 |
| Validation | D_dimer | Anti_CCP | -0.001 | -0.001 to 0.0 | 0.07074 |
| Validation | D_dimer | IgG | 0.024 | -0.017 to 0.065 | 0.2475 |
| Validation | D_dimer | IgA | -0.074 | -0.187 to 0.039 | 0.1997 |
| Validation | D_dimer | IgM | -0.12 | -0.324 to 0.084 | 0.2497 |
| Validation | D_dimer | C3 | -0.179 | -0.975 to 0.617 | 0.6587 |
| Validation | D_dimer | C4 | -0.201 | -2.2 to 1.798 | 0.8435 |
| Validation | D_dimer | Age | 0.008 | -0.002 to 0.018 | 0.09799 |
| Validation | D_dimer | Gender_Male | 0.114 | -0.162 to 0.39 | 0.4178 |
| Validation | D_dimer | ESR | 0.019 | 0.012 to 0.026 | 3.454e-08 |
| Validation | D_dimer | hsCRP | 0.012 | 0.007 to 0.016 | 3.541e-08 |
| Validation | Fibrinogen | const | 1.474 | 1.065 to 1.883 | 3.384e-12 |
| Validation | Fibrinogen | RF | 0.0 | -0.0 to -0.0 | 2.925e-06 |
| Validation | Fibrinogen | Anti_CCP | 0.0 | -0.0 to 0.0 | 0.6415 |
| Validation | Fibrinogen | IgG | -0.024 | -0.04 to -0.009 | 0.002221 |
| Validation | Fibrinogen | IgA | 0.012 | -0.031 to 0.056 | 0.5732 |
| Validation | Fibrinogen | IgM | 0.009 | -0.068 to 0.087 | 0.8119 |
| Validation | Fibrinogen | C3 | 1.288 | 0.984 to 1.592 | 3.883e-16 |
| Validation | Fibrinogen | C4 | 0.892 | 0.128 to 1.655 | 0.02214 |
| Validation | Fibrinogen | Age | 0.005 | 0.001 to 0.009 | 0.009784 |
| Validation | Fibrinogen | Gender_Male | 0.283 | 0.178 to 0.389 | 1.689e-07 |
| Validation | Fibrinogen | ESR | 0.015 | 0.012 to 0.018 | 2.5590000000000002e-28 |
| Validation | Fibrinogen | hsCRP | 0.006 | 0.004 to 0.007 | 7.349e-13 |

*Regression coefficients (β) represent the estimated change in each coagulation parameter per unit increase in the corresponding immunological marker, as measured. Models were adjusted for age, sex, erythrocyte sedimentation rate, and high-sensitivity C-reactive protein.

Supplementary Table 3. Comparison of unadjusted and adjusted regression models evaluating immune–coagulation associations..

| Outcome | Predictor | Beta (NoCov) | 95% CI (NoCov) | P (NoCov) | Beta (Adj) | 95% CI (Adj) | P (Adj) |
| --- | --- | --- | --- | --- | --- | --- | --- |
| INR | RF | 0.000 | -0.000 to 0.000 | 0.176 | -0.000 | -0.000 to 0.000 | 0.146 |
| INR | Anti_CCP | 0.000 | -0.000 to 0.000 | 0.307 | 0.000 | -0.000 to 0.000 | 0.080 |
| INR | IgG | 0.004 | 0.003 to 0.005 | 3.911e-09 | 0.003 | 0.002 to 0.004 | 2.569e-06 |
| INR | IgA | 0.001 | -0.003 to 0.004 | 0.676 | -0.001 | -0.004 to 0.003 | 0.705 |
| INR | IgM | -0.002 | -0.009 to 0.005 | 0.658 | 0.001 | -0.006 to 0.008 | 0.852 |
| INR | C3 | -0.004 | -0.029 to 0.020 | 0.721 | -0.050 | -0.076 to -0.025 | 0.000 |
| INR | C4 | -0.057 | -0.122 to 0.008 | 0.084 | -0.014 | -0.076 to 0.048 | 0.654 |
| PT | RF | 0.000 | -0.000 to 0.000 | 0.665 | -0.000 | -0.000 to 0.000 | 0.405 |
| PT | Anti_CCP | -0.001 | -0.001 to -0.000 | 0.002 | -0.001 | -0.001 to -0.000 | 0.007 |
| PT | IgG | 0.056 | 0.037 to 0.075 | 7.593e-09 | 0.066 | 0.046 to 0.086 | 1.192e-10 |
| PT | IgA | -0.027 | -0.082 to 0.028 | 0.335 | -0.016 | -0.072 to 0.040 | 0.573 |
| PT | IgM | 0.001 | -0.104 to 0.107 | 0.983 | 0.045 | -0.062 to 0.152 | 0.413 |
| PT | C3 | -0.307 | -0.673 to 0.058 | 0.099 | -0.365 | -0.756 to 0.025 | 0.067 |
| PT | C4 | 0.707 | -0.253 to 1.667 | 0.149 | 0.850 | -0.104 to 1.805 | 0.081 |
| APTT | RF | -0.000 | -0.001 to 0.000 | 0.051 | -0.001 | -0.001 to -0.000 | 0.006 |
| APTT | Anti_CCP | -0.004 | -0.005 to -0.003 | 3.355e-09 | -0.004 | -0.005 to -0.003 | 1.369e-08 |
| APTT | IgG | 0.183 | 0.116 to 0.251 | 1.137e-07 | 0.202 | 0.131 to 0.274 | 2.771e-08 |
| APTT | IgA | -0.184 | -0.379 to 0.011 | 0.065 | -0.153 | -0.353 to 0.048 | 0.135 |
| APTT | IgM | 0.190 | -0.187 to 0.566 | 0.323 | 0.236 | -0.146 to 0.620 | 0.226 |
| APTT | C3 | -1.938 | -3.240 to -0.637 | 0.004 | -2.180 | -3.577 to -0.783 | 0.002 |
| APTT | C4 | 5.816 | 2.399 to 9.233 | 0.001 | 6.255 | 2.841 to 9.669 | 0.000 |
| D_dimer | RF | 0.000 | 0.000 to 0.000 | 0.003 | -0.000 | -0.000 to 0.000 | 0.410 |
| D_dimer | Anti_CCP | -0.001 | -0.001 to -0.000 | 0.010 | -0.001 | -0.001 to -0.000 | 0.048 |
| D_dimer | IgG | 0.107 | 0.079 to 0.136 | 2.740e-13 | 0.061 | 0.033 to 0.088 | 1.784e-05 |
| D_dimer | IgA | 0.079 | -0.003 to 0.162 | 0.059 | -0.050 | -0.128 to 0.028 | 0.211 |
| D_dimer | IgM | -0.221 | -0.380 to -0.062 | 0.007 | -0.111 | -0.260 to 0.038 | 0.144 |
| D_dimer | C3 | 1.577 | 1.027 to 2.127 | 2.166e-08 | -0.166 | -0.708 to 0.377 | 0.549 |
| D_dimer | C4 | -1.489 | -2.933 to -0.045 | 0.043 | -0.230 | -1.556 to 1.096 | 0.734 |
| PT_Activity | RF | -0.001 | -0.002 to 0.000 | 0.098 | 0.000 | -0.001 to 0.001 | 0.346 |
| PT_Activity | Anti_CCP | -0.005 | -0.009 to -0.002 | 0.002 | -0.006 | -0.010 to -0.003 | 8.852e-05 |
| PT_Activity | IgG | -0.604 | -0.783 to -0.425 | 4.732e-11 | -0.530 | -0.711 to -0.349 | 1.049e-08 |
| PT_Activity | IgA | -0.424 | -0.941 to 0.094 | 0.108 | -0.307 | -0.817 to 0.203 | 0.238 |
| PT_Activity | IgM | 0.489 | -0.509 to 1.487 | 0.337 | 0.184 | -0.790 to 1.157 | 0.711 |
| PT_Activity | C3 | 1.645 | -1.806 to 5.096 | 0.350 | 7.200 | 3.649 to 10.751 | 7.286e-05 |
| PT_Activity | C4 | 2.069 | -6.994 to 11.132 | 0.654 | -3.745 | -12.425 to 4.934 | 0.397 |
| Fibrinogen | RF | 0.000 | -0.000 to 0.000 | 0.133 | -0.000 | -0.000 to -0.000 | 1.408e-09 |
| Fibrinogen | Anti_CCP | -0.000 | -0.000 to 0.000 | 0.408 | 0.000 | -0.000 to 0.000 | 0.489 |
| Fibrinogen | IgG | 0.013 | 0.001 to 0.026 | 0.037 | -0.021 | -0.031 to -0.011 | 4.182e-05 |
| Fibrinogen | IgA | 0.110 | 0.073 to 0.147 | 4.198e-09 | 0.007 | -0.021 to 0.036 | 0.612 |
| Fibrinogen | IgM | -0.087 | -0.158 to -0.017 | 0.015 | 0.002 | -0.052 to 0.056 | 0.952 |
| Fibrinogen | C3 | 2.559 | 2.315 to 2.803 | 4.671e-84 | 1.347 | 1.149 to 1.545 | 9.052e-39 |
| Fibrinogen | C4 | 0.111 | -0.528 to 0.751 | 0.733 | 0.970 | 0.487 to 1.453 | 8.520e-05 |

*Changes in effect estimates after adjustment for demographic and inflammatory covariates are shown to assess the robustness of associations. This table is intended to illustrate the influence of potential confounders rather than to infer causality.

Supplementary Table 4. Sensitivity analyses of immune–coagulation associations using alternative modeling strategies, including reduced-variable models and principal component analysis (PCA)–based models.

| Outcome | Predictor | Beta (Model1) | P (Model1) | Beta (Model2) | P (Model2) | Beta (Model3) | P (Model3) |
| --- | --- | --- | --- | --- | --- | --- | --- |
| INR | RF | -0.000 | 0.146 | -0.000 | 0.146 | nan | nan |
| INR | Anti_CCP | 0.000 | 0.080 | 0.000 | 0.080 | nan | nan |
| INR | IgG | 0.003 | 2.569e-06 | 0.003 | 2.569e-06 | nan | nan |
| INR | IgA | -0.001 | 0.705 | -0.001 | 0.705 | nan | nan |
| INR | IgM | 0.001 | 0.852 | 0.001 | 0.852 | nan | nan |
| INR | C3 | -0.050 | 0.000 | -0.050 | 0.000 | nan | nan |
| INR | C4 | -0.014 | 0.654 | -0.014 | 0.654 | nan | nan |
| PT | RF | -0.000 | 0.405 | -0.000 | 0.405 | nan | nan |
| PT | Anti_CCP | -0.001 | 0.007 | -0.001 | 0.007 | nan | nan |
| PT | IgG | 0.066 | 1.192e-10 | 0.066 | 1.192e-10 | nan | nan |
| PT | IgA | -0.016 | 0.573 | -0.016 | 0.573 | nan | nan |
| PT | IgM | 0.045 | 0.413 | 0.045 | 0.413 | nan | nan |
| PT | C3 | -0.365 | 0.067 | -0.365 | 0.067 | nan | nan |
| PT | C4 | 0.850 | 0.081 | 0.850 | 0.081 | nan | nan |
| APTT | RF | -0.001 | 0.006 | -0.001 | 0.006 | nan | nan |
| APTT | Anti_CCP | -0.004 | 1.369e-08 | -0.004 | 1.369e-08 | nan | nan |
| APTT | IgG | 0.202 | 2.771e-08 | 0.202 | 2.771e-08 | nan | nan |
| APTT | IgA | -0.153 | 0.135 | -0.153 | 0.135 | nan | nan |
| APTT | IgM | 0.236 | 0.226 | 0.236 | 0.226 | nan | nan |
| APTT | C3 | -2.180 | 0.002 | -2.180 | 0.002 | nan | nan |
| APTT | C4 | 6.255 | 0.000 | 6.255 | 0.000 | nan | nan |
| INR | AIC/BIC | -3482.713 | -3418 | -3482.713 | -3418 | -3433.317 | -3396 |
| PT | AIC/BIC | 5417.268 | 5482 | 5417.268 | 5482 | 5459.169 | 5497 |
| APTT | AIC/BIC | 9566.713 | 9631 | 9566.713 | 9631 | 9602.539 | 9640 |

*PCA-based models were applied to immunological predictors as composite components; therefore, regression coefficients for individual biomarkers are not applicable in PCA models and are reported as not available. Model fit indices (AIC and BIC) are provided to facilitate comparison of overall model performance across sensitivity analyses.
