# Supplementary material for: A large-scale profiling study of immune–coagulation associations in rheumatoid arthritis
Source: Front Immunol. 2026 Mar 17;17:1789560. doi: 10.3389/fimmu.2026.1789560 (PMC13047912; doi:10.3389/fimmu.2026.1789560)
Supplement: Supplementary file 2 [file DataSheet1.docx]

Supplementary Figure 1. Correlation matrices illustrating pairwise associations between immunological markers and coagulation parameters in patients with rheumatoid arthritis.


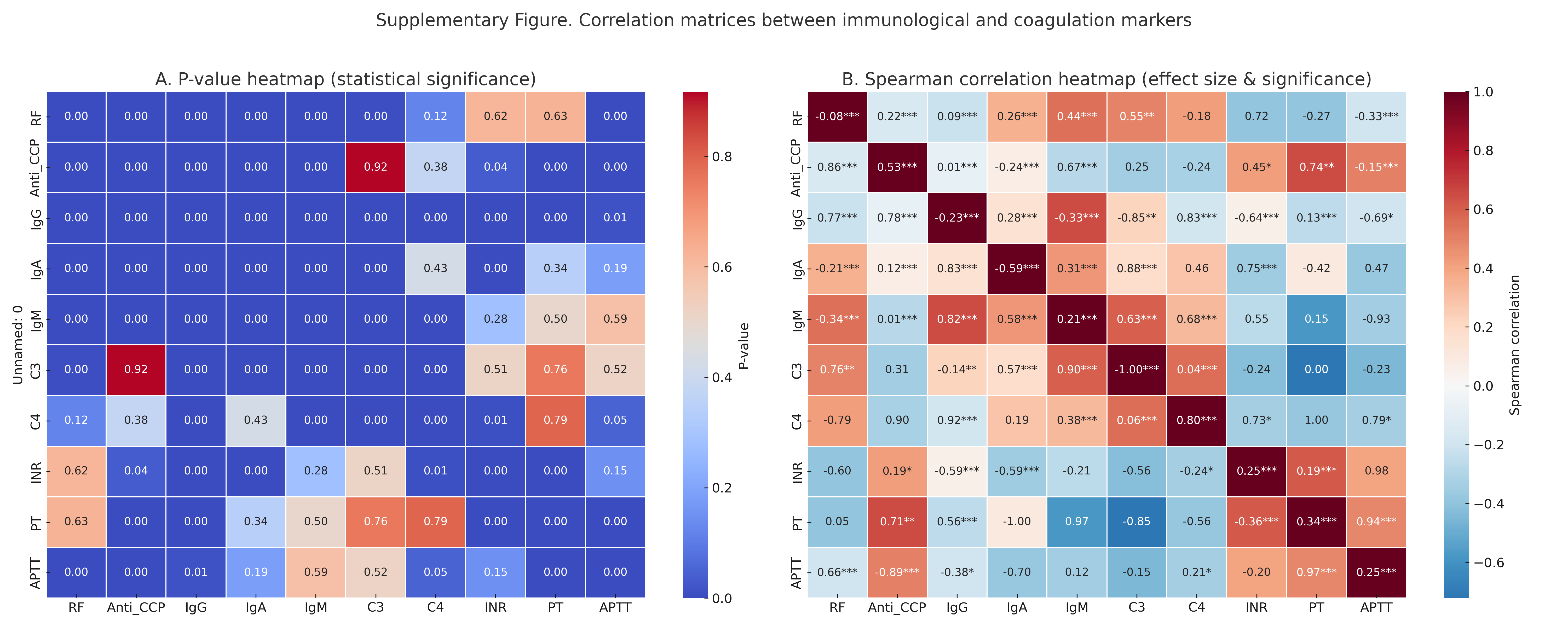


1. Heatmap of P values derived from Spearman correlation analyses.
   B. Heatmap of Spearman correlation coefficients (ρ), with color intensity representing effect size and asterisks indicating nominal statistical significance (*P < 0.05; **P < 0.01; ***P < 0.001).

Correlation analyses are presented for exploratory purposes to visualize the overall association structure and to inform subsequent multivariable regression modeling. Statistical significance in this figure does not imply independence, causality, or clinical relevance.
