# Supplementary material for: A large-scale profiling study of immune–coagulation associations in rheumatoid arthritis
Source: Front Immunol. 2026 Mar 17;17:1789560. doi: 10.3389/fimmu.2026.1789560 (PMC13047912; doi:10.3389/fimmu.2026.1789560)

Supplementary Figure 2. Comparison of regression coefficients across sensitivity models for validated associations between immunological markers and coagulation parameters in rheumatoid arthritis. Bars represent the estimated regression coefficient (β) for each association under three modeling approaches: Model 1 (full model including all immunological markers), Model 2 (reduced model excluding variables with high variance inflation factor), and Model 3 (principal component analysis-based model using the first two principal components to replace immunological markers). A vertical dashed line at β = 0 indicates no association. Consistency in effect direction and magnitude across models indicates robustness of the associations to potential multicollinearity and model specification differences.


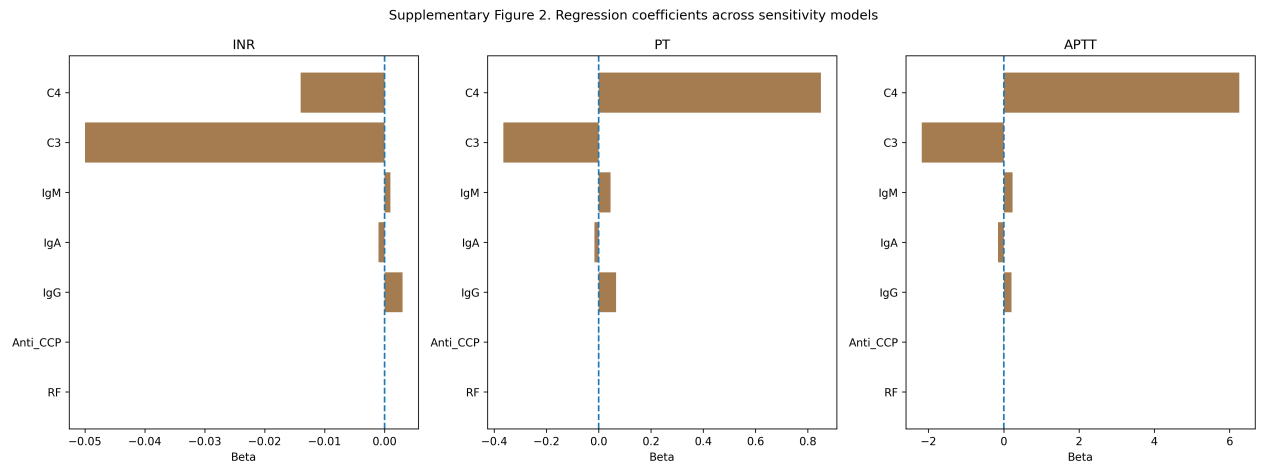

Supplement: Supplementary file 3 [file DataSheet2.docx]
